# Protective Effect of Cytosolic Phospholipase A2 Inhibition against Inflammation and Degeneration by Promoting Regulatory T Cells in Rats with Experimental Autoimmune Encephalomyelitis

**DOI:** 10.1155/2014/890139

**Published:** 2014-03-23

**Authors:** Dan Yang, Hong-Fei Ji, Xue-Mei Zhang, Hui Yue, Lin Lin, Yu-Yan Ma, Xiang-nan Huang, Jin Fu, Wei-Zhi Wang

**Affiliations:** ^1^Department of Neurology, Second Affiliated Hospital, Harbin Medical University, 246 Xuefu Road, Nangang District, Harbin 150086, China; ^2^Department of Molecular Biology and Genetic Engineering, Cancer Institute of Heilongjiang Province, Harbin Medical University, Harbin 150081, China

## Abstract

Cytosolic phospholipase A2 (cPLA_2_) is the rate-limiting enzyme that initiates the production of various inflammatory mediators. Previous studies have shown that inhibiting cPLA_2_ exerts a neuroprotective effect on experimental autoimmune encephalomyelitis (EAE) by ameliorating the severity of the disease and influencing Th1 and Th17 responses. However, it remains unclear whether treatment with a cPLA_2_ inhibitor will influence the regulatory T cells (Tregs) that play a critical role in maintaining immune homeostasis and preventing autoimmune diseases. In this study, the cPLA_2_ inhibitor AX059 reduced the onset and progression of EAE in Lewis rats. In addition, this effect was accompanied by activation of Tregs and alterations in the expression of their various cytokines. The study therefore demonstrated that Tregs are involved in the immunomodulatory effect mediated by cPLA_2_ inhibition. These findings may have clinical application in the treatment of multiple sclerosis.

## 1. Introduction

Multiple sclerosis (MS) is an inflammatory, demyelinating disorder of the central nervous system (CNS) affecting over 2.5 million young adults worldwide. Much of our understanding of the pathogenesis of MS is based on studies using the animal model, experimental autoimmune encephalomyelitis (EAE) [1]. Many variations of EAE are now available; for example, Lewis rats can develop monophasic or chronic EAE after injection of myelin basic protein (MBP), and this is a stable animal model to explore the mechanisms underlying CNS autoimmune diseases [2, 3].

The mechanisms underlying the pathogenesis of MS or EAE were previously believed to be mediated mainly by Th1 cells and Th1-related cytokines, which initiate a pathogenic response directed against the components of CNS myelin and lead to inflammation, demyelination, axonal damage, and, ultimately, functional deficits [4]. Nevertheless, many recent reports have suggested that abnormal regulatory T cells (Tregs) are involved in the pathogenesis of autoimmune demyelination in EAE and MS [5, 6]. Tregs are a subpopulation of CD4+ T cells which are central to the acquisition and maintenance of immunological self-tolerance, as well as tolerance of tissue grafts and prevention of autoimmune diseases [7]. CD25 has been identified as a phenotypic marker for Tregs, and the forkhead/winged helix transcription factor forkhead box P3 (Foxp3) is its specific transcription factor. In patients with MS, a functional defect of Tregs has been found [8]. In EAE, Tregs administered to mice can also significantly reduce EAE severity [9, 10] and have been shown to accumulate within the CNS during recovery [11].

In addition to autoreactive T cells and inflammatory cytokines, various other mediators of inflammation that recruit and modulate immune cells play major roles in the pathogenesis of these disorders. Phospholipase A2 (PLA_2_) is a heterogeneous group of enzymes that specifically hydrolyze fatty acids at the sn-2 position of cell membrane phospholipids, which gives rise to eicosanoids that contribute to various aspects of inflammation through cyclooxygenase (COX) and lipoxygenase pathways [12, 13]. Among the various PLA_2_ enzymes, cytosolic PLA2 (cPLA_2_) is the predominant isoform with enzymatic activity, and cPLA_2_ plays important roles in the arachidonic acid cascade [14]. Previous studies have suggested that cPLA_2_ participates in EAE development and that inhibition of cPLA_2_ may be valuable for the prevention EAE, which suggests that cPLA_2_ inhibitors may be potentially useful immunomodulators in the treatment of MS [15, 16]. Furthermore, one study in a mouse EAE model has shown that the involvement of cPLA_2_ in the pathogenesis of EAE is associated with Th17-type responses [17]. However, there are no reports describing the correlation of cPLA_2_-mediated neuroinflammation and neurodegeneration with Tregs.

In the present study, we investigated for the first time the effect of cPLA_2_ in rats with EAE by using a selective cPLA_2_ inhibitor. Our findings indicate that blocking cPLA_2_ can reduce the onset and progression of the condition. In addition, we found that Tregs and their regulatory cytokines are altered when cPLA_2_ is blocked in rats with EAE.

## 2. Materials and Methods

### 2.1. Animals

Female Lewis rats (8 weeks old) weighing approximately 160–170 g were obtained from Beijing Vital River Laboratory Animal Co. Ltd (Beijing, China). All rats were bred in specific pathogen-free and climate-controlled conditions. All in vivo experiments in these animals were performed in accordance with the Committee on Ethics of Animal Experiments, Harbin Medical University.

### 2.2. EAE Induction

EAE was induced as previously described [18]. Briefly, rats were immunized with 25 *μ*g of myelin basic protein MBP (Sigma) emulsified with complete Freund's adjuvant (CFA) containing 5 mg/mL of* Mycobacterium butyricum *(Sigma). This solution (100 *μ*L) was injected subcutaneously into the footpad of the animals' hind limbs. Rats injected with the vehicle solution were used as control. The rats were evaluated daily for weight loss and scored for neurological impairment as follows: 0 = no signs; 1 = loss of tonicity of the distal portion of the tail; 2 = total loss of tail tonicity; 3 = hind limb weakness (partial paralysis); 4 = complete hind limb paralysis and urinary incontinence; 5 = dead.

### 2.3. Drug Treatment

Rats were randomly assigned to either the treatment or control group and were treated with either the 2-oxoamide cPLA_2_ inhibitor AX059 (intraperitoneal injections of 200 *μ*L of a 4 mmol/L solution) or vehicle (PBS containing 1% dimethyl sulfoxide) administered once daily for 2 weeks, starting on the day of immunization for EAE induction. The synthesis of AX059 was in accordance with the method of Stephens et al. [19] and Barbayianni et al. [20]. Monitoring was performed on a blinded basis so that the person doing the scoring was unaware of the experimental group that the animals had been assigned to.

### 2.4. Histological Analysis

Rats were deeply anesthetized by inhalation of 5% carbon dioxide on day 14. After euthanasia and blood withdrawal, brain and spinal cord samples were dissected and fixed in 4% paraformaldehyde before embedding in paraffin. Selected panels of serial sections (4-5 *μ*m) were processed for routine hematoxylin and eosin (H&E) staining, Luxol fast blue staining, and immunohistochemical labeling with anti-Foxp3 and analyzed with a Nikon microscope. The number of inflammatory foci containing at least 20 cells was counted in each H&E-stained section in a blinded fashion by the same pathologist.

### 2.5. Flow Cytometry Analysis

To analyze the prevalence of Tregs, cellular surface markers were evaluated via flow cytometry using the following antibodies: fluorescein isothiocyanate (FITC) conjugated CD4 and phycoerythrin (PE) conjugated CD25 (all purchased from BD Biosciences Pharmingen, San Diego, CA). Lymph cells were washed with cold phosphate-buffered saline (PBS) and resuspended in further 100 mL of PBS. Cells were stained for 20 min at 4°C with FITC-CD4 and PE-CD25 antibodies. Staining was visualized on an FACS Calibur flow cytometer (BD, Franklin Lakes, NJ, USA) with Cell Quest (version 3.2.1f1, BD).

### 2.6. Assessment of Cytokine Production

To assess cytokine production, spleens were isolated from rats in each treatment group. Mononuclear cell suspensions were prepared and 2 × 10^6^ cells were cultured in complete RPMI medium and initiation with MBP. The cells were then treated with either AX059 (30 *μ*mol/L) or vehicle for 60 hours. Supernatant obtained from in vitro cultures was analyzed for interleukin-6 (IL-6), interleukin-10 (IL-10), and transforming growth factor-*β* (TGF-*β*) according to the manufacturer's protocol (BD Bioscience). For the FACS experiment, lymphocytes were used.

### 2.7. Statistical Analysis

Data were expressed as means ± standard deviation, and statistical differences in the severity of neurological impairment, inflammatory foci, Foxp3 expression, flow cytometry data, and cytokine production between the various groups were determined by analysis of variance (ANOVA) and the Mann-Whitney rank-sum test. Statistical analyses were performed using SPSS for Windows (version 11.0, SPSS Inc., Chicago, USA). A *P* value less than 0.05 was considered statistically significant.

## 3. Results

### 3.1. Effect of cPLA_2_ Inhibition on the Severity of EAE in Lewis Rats

**Figure 1:**
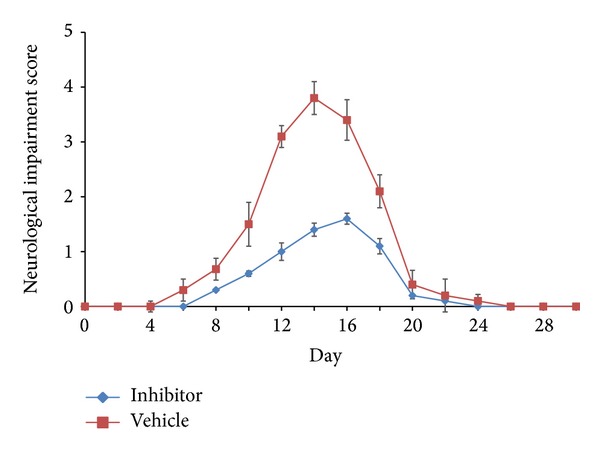
Neurological impairment scores in Lewis rats with experimental autoimmune encephalomyelitis (EAE). The rats were immunized with 25 *μ*g of myelin basic protein (MBP) and 5 mg/mL of* Mycobacterium butyricum* and then treated with either the cPLA_2_ inhibitor AX059 or vehicle once daily, starting on the day of immunization for EAE induction. EAE development in the rats was assessed on a scale of 0–5.

To investigate the effect of the cPLA_2_ inhibitor AX059 on the development of EAE, Lewis rats were treated separately with AX059 and vehicle. The rats treated with AX059 were resistant to EAE induction; 4 of 12 rats in this group developed EAE as compared with all 12 rats treated with vehicle. In addition, the average maximum neurological impairment score was significantly reduced when compared with the vehicle treatment group, the peak neurological impairment occurred 2 days later, and the remaining neurological impairment time was shorter, indicating that inhibition of cPLA_2_ delayed the onset of EAE and hastened its recovery (Figure 1).

To further assess inflammation in the central nervous system, the pathological changes revealed by H&E staining of the animals' brains and spinal cords were consistent with the neurological impairment scores. Inflammatory cells infiltrated around blood vessels in the leptomeninges and white matter and generated several focal lesions of inflammation that were related to the severity of EAE. Pathological changes in the spinal cord were more marked than those in the brain.

**Figure 2:**
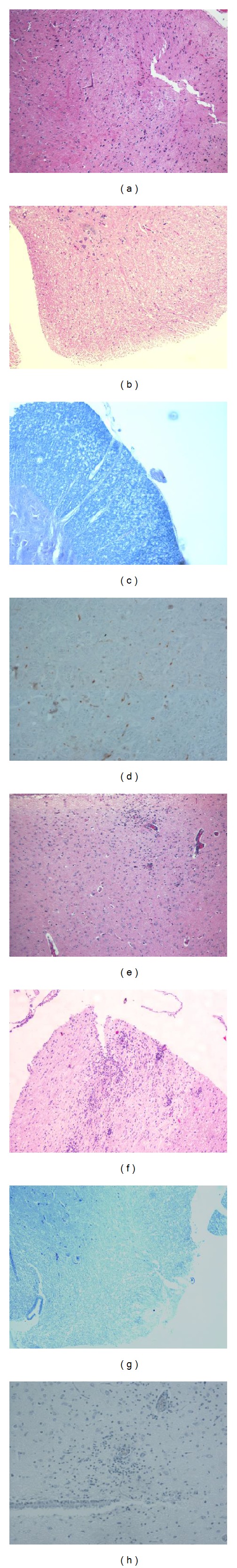
Treatment with the cPLA_2_ inhibitor AX059 reduced brain and spinal cord damage in EAE induced in Lewis rats. The figures shows hematoxylin and eosin (H&E)-stained sections from AX059-treated ((a) brain; (b) spinal cord) and vehicle-treated ((e) brain; (f) spinal cord) rats; spinal cord sections from AX059-treated (c) or vehicle-treated (g) rats stained with Luxol fast blue; and Foxp3 expression in spinal cord sections from AX059-treated (d) or vehicle-treated (h) rats by immunohistochemical staining.

**Figure 3:**
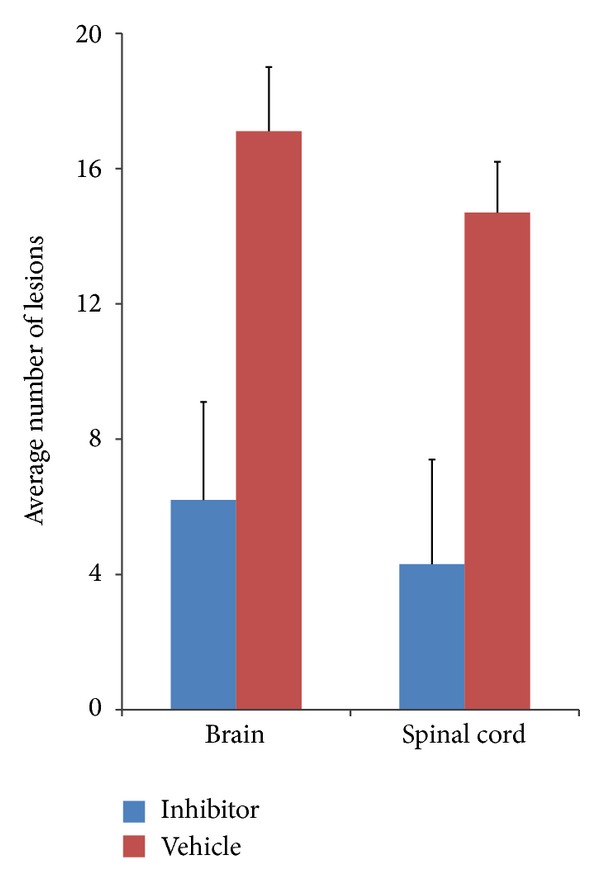
Morphometric analysis of infiltrated lesions in the brains and spinal cords of AX059-treated and vehicle-treated rats.

Demyelination was also evaluated in Luxol fast blue sections of the spinal cord. Although obvious signs of demyelination within white matter were observed in vehicle-treated rats, only minimal changes were observed in AX059-treated rats (Figure 2). Microscopic examination found that there were 17.2 ± 2.7 and 14.7 ± 2.1 focal lesions in AX059-treated rats' brains and spinal cords, respectively, whereas 7.1 ± 1.9 and 4.3 ± 1.5 in rats treated separately with vehicle. The number of focal lesions in AX059-treated rats was significantly less than in vehicle-treated rats (Figure 3).

### 3.2. Effect of cPLA_2_ Inhibition on Tregs

**Figure 4:**
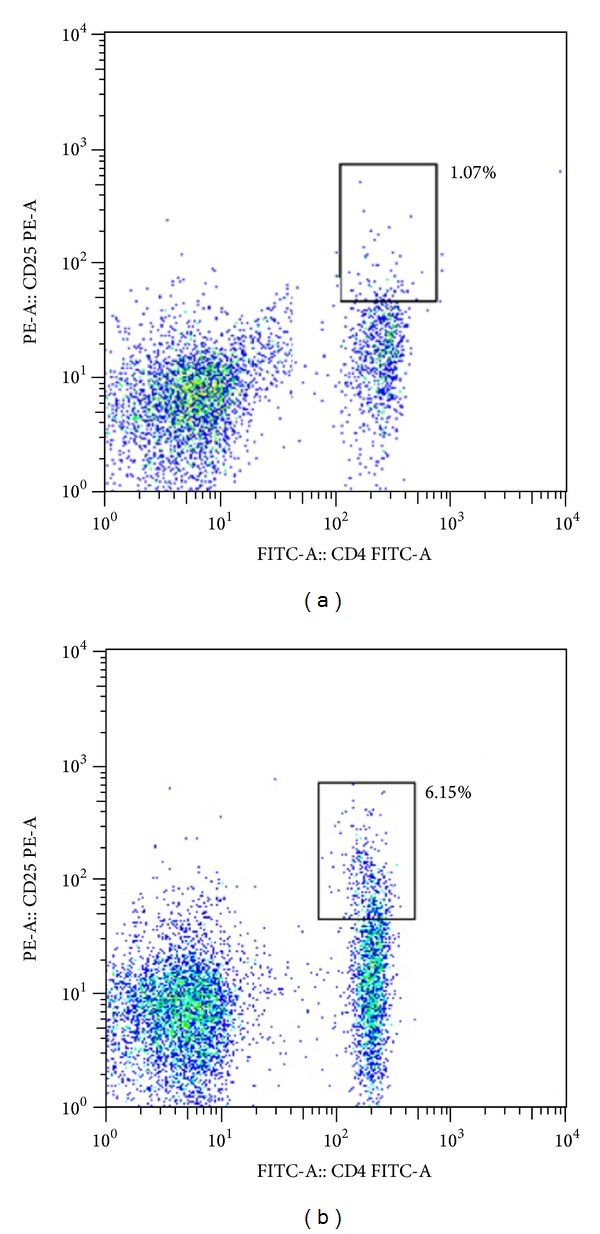
Rats treated with AX059 (b) displayed an increase in the percentage of Tregs during EAE induction compared with those treated with vehicle (a).

To analyze the effect of cPLA_2_ inhibitor treatment on Tregs, monocytes were isolated from the spleens of rats in the 2 treatment groups at 14 days, and expression levels of CD4 and CD25 on the cells' surfaces were measured by flow cytometry. Compared with rats in the vehicle-treated group, AX059-treated rats showed markedly increased Tregs, and the difference in the percentages of Tregs (5.9 ± 1.0% versus 1.3 ± 0.7%) between the 2 groups (Figure 4) reached statistical significance (*P* < 0.05).

To further investigate functional changes of Tregs in the lesions, including both the brain and spinal cord, we used an immunocytochemical method to assess the expression of Foxp3, which is critical for the regulatory function of Tregs and is also a unique marker for their identification. As shown in Figure 2, Tregs were located in both the white and grey matter of the brain and spinal cord and were present in larger numbers in rats treated with AX059 than in vehicle-treated rats (Figures 2(d) and 2(h)). There was little infiltration of T cells and minimal myelin and axonal damage in the location of Tregs homing.

### 3.3. Effect of cPLA_2_ Inhibition on the Cytokine Expression of Tregs

**Figure 5:**
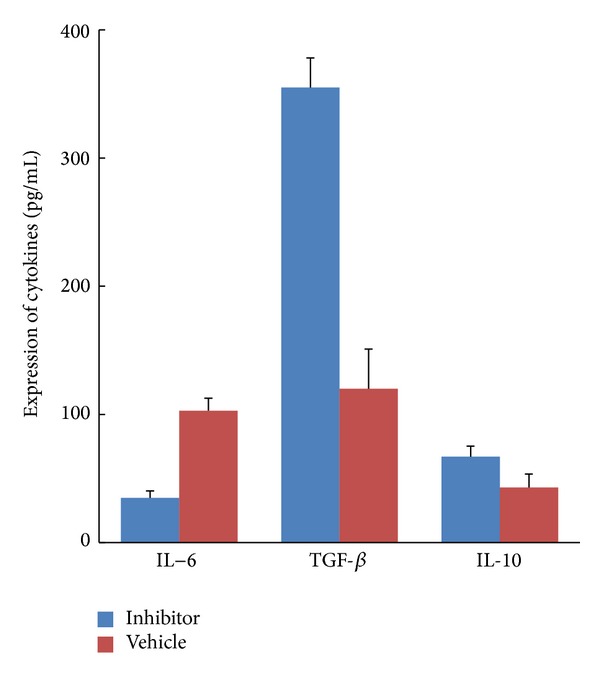
The cPLA_2_ inhibitor AX059 increased the expression of TGF-*β* and IL-10 and downregulated the expression of IL-6 in the rats' spleen cells.

To explore the mechanisms underlying the effect of cPLA_2_ inhibition on Tregs, we analyzed 3 cytokines, including TGF-*β*, IL-10, and IL-6, which are closely associated with the functions of Tregs. The immunosuppressive cytokines TGF-*β* and IL-10 were increased in AX059-treated rats with EAE as compared with vehicle-treated rats. Conversely, a striking reduction was observed in the typical inflammatory cytokine IL-6 (Figure 5). Together, these findings indicate that AX059 induced the activation and proliferation of Tregs to interfere with the pathological immune response. The data suggest that changes in these 3 cytokines play a pivotal role in the induction of Tregs by AX059 and subsequently in the diminished infiltration of inflammatory cells and the neuroprotection afforded by AX059.

## 4. Discussion

As the key esterase involved in the synthesis of many inflammatory mediators, cPLA_2_ has been known to play extensive roles in autoimmune and oxidative stress in neurological disorders [21]. Recent studies have demonstrated that cPLA_2_ serves as a central mediator of EAE and MS development, the potential mechanism of which includes not only initiating the secretion of inflammatory effectors and chemokines, but also promoting immune cell infiltration, demyelination, and axonal loss [22, 23]. cPLA_2_-deficient mice are known to be resistant to EAE, and inhibition of cPLA_2_ exerts an anti-inflammatory effect in mice with EAE by delaying or reducing the onset and progression of the disease [24]. Therefore, cPLA_2_ is believed to be important in the pathogenesis of MS, and blockade of the enzyme might have therapeutic benefits in reducing its progression.

Rat strains differ from mice in their susceptibility to the disease, which gives us another insight into human MS. In the present study, we showed that the cPLA_2_ inhibitor AX059 played a protective role in the Lewis rat EAE model, decreasing the incidence, delaying the peak of neurological impairment, and reducing the severity of the condition. Furthermore, treatment with AX059 induced activation of Tregs and enhanced their numbers. Our findings therefore suggest that Tregs are involved in the anti-inflammatory effect of cPLA_2_ inhibition in the rat EAE model.

Previous studies have shown that cPLA_2_ has an important role in the regulation of T cell differentiation during EAE development and that blocking cPLA_2_ with an inhibitor or by gene knockout prevents inflammatory cell production [17, 25, 26]. Marusic et al. reported that cPLA_2*α*_^−/−^ mice develop impaired Th1-type responses [24], while another study revealed that blockade of cPLA_2_ led to diminished Th1-type and Th17-type cytokines, as well as striking reductions in interferon- (IFN-) *γ*, tumor necrosis factor (TNF) and IL-17 concentrations [17]. Taketomi et al. also showed that cPLA_2_ drives mast cell maturation in anaphylaxis [27]. Based on these findings and the fact that cPLA_2_ inhibition was closely associated with multiple aspects of EAE pathogenesis and various types of T cell responses in the present study, it is worth determining whether treatment with a cPLA_2_ inhibitor will alter the abundance and function of Tregs, which are the key regulators of lymphocyte proliferation and differentiation.

In our study, an increase in the percentage of Tregs was observed in Lewis rats with EAE after treatment with AX059 in comparison with rats treated with vehicle by using flow cytometry. To further evaluate whether the Tregs exhibited an immunosuppressive effect, we assessed the expression of Foxp3, a specific transcription factor that exerts immunosuppressive functions. Brain and spinal cord lesions were infiltrated by significantly higher numbers of Tregs in AX059-treated rats compared with vehicle-treated rats, and activated Tregs were induced by the inhibition of cPLA_2_. To our knowledge, this is the first study to demonstrate an association between Tregs and cPLA_2_ inhibition. In agreement with our findings, treatments that ameliorate the course of EAE have been found to enhance the development of Tregs during recovery in mouse EAE models [28–30]. Furthermore, some immunomodulatory therapies approved for MS such as glucocorticoids, interferon-*β*, and glatiramer acetate have been found to have a significant effect on Tregs [16, 31–33]. Thus, our findings may have clinical application in the treatment of MS.

The development and maintenance of the anti-inflammatory effects of Tregs are regulated by several cytokines, including TGF-*β*, IL-6, and IL-10 [34]. The development of Tregs is correlated with enhanced TGF-*β* expression and reduced levels of IL-6. In addition to inducing differentiation and development of Tregs, TGF-*β* also can prolong the half-life of Foxp3 RNA species and phosphorylate chromatin-bound Foxp3 [35], and IL-6 plays a crucial role in regulating the balance between Treg cells and Th17 cells, which are involved in the pathogenesis of EAE and MS [36, 37]. At the same time, IL-6 can destroy the immunosuppressive function of Treg cells under inflammatory conditions [38, 39]. Murai et al. reported that IL-10 was required to maintain a Treg-suppressive function and to maintain expression of the Foxp3 transcription factor [40]. Most recently, Klose reported that IL-10 production of transduced neural stem/progenitor cells (NSPC^IL-10^) ameliorates the clinical disease course of EAE, and the therapeutic mechanism was that NSPC^IL-10^ suppressed IL-2 and IFN-*γ* production and did not induce a higher percentage of Tregs since IL-10 was produced in NSPC instead of lymphocyte [41]. On the other hand, previous studies have shown that cPLA regulates the production of cytokines, and blockade of cPLA_2_ causes suppression of Th1-type and Th17-type cytokines [15, 17]. Thus, it is conceivable that cPLA_2_ inhibition might play a central role by regulating the cytokine expression of Tregs. To test this hypothesis, we analyzed the expression of TGF-*β*, IL-6, and IL-10. When the cells were exposed to AX059, the positive modulatory effect of TGF-*β* and IL-10 on Tregs differentiation increased. In contrast, the negative modulatory effect of IL-6 was significantly reduced. Previous studies have revealed that, whereas cPLA_2_ inhibition impairs the induction of Th1-type and Th17-type cytokines, no report mentioned that it also makes contribution to increasing the production of Tregs-related cytokines.

In conclusion, our study in a rat model has demonstrated that cPLA_2_ may be involved in onset and progression of EAE and that cPLA_2_ inhibitor treatment may offer beneficial effects in reducing disease severity by regulating Tregs and mediators of their effects such as various cytokines and other T cells. Therefore, the development of selective cPLA_2_ inhibitors may provide hope for the treatment of human CNS autoimmune disease.
